# Indocyanine Green Fluorescence Versus Blue Dye or Radioisotope Regarding Detection Rate of Sentinel Lymph Node Biopsy and Nodes Removed in Breast Cancer: A Systematic Review and Meta-Analysis

**DOI:** 10.31557/APJCP.2020.21.5.1187

**Published:** 2020-05

**Authors:** Sarun Thongvitokomarn, Nuanphan Polchai

**Affiliations:** 1 *Department of Surgery, Panyananthaphikkhu Chonprathan Medical center, Srinakharinwirot University, Nonthaburi, Thailand. *; 2 *Division of Head Neck and Breast Surgery, Department of Surgery, Faculty of Medicine, Siriraj Hospital, Mahidol University, Bangkok, Thailand. *

**Keywords:** Indocyanine green, blue dye, radioisotope, sentinel lymph node, breast cancer, meta-analysis

## Abstract

**Background:**

Either blue dye (BD) or radioisotope (RI) is mainly used for sentinel lymph node biopsy (SLNB) in breast cancer patients. Unlike the BD, RI has lower false-negative rate of SLNB. However, its lymphoscintigraphy, difficulty in preoperative injection, and undetected sentinel lymph nodes in some cases cause surgeons to rely only on BD. Currently, indocyanine green (ICG) fluorescence method (ICG-SLNB) is increasingly used as an alternative to the conventional mapping methods in many centers. This systematic review compared ICG with the conventional method of BD or RI in terms of detection rate of SLNB and the number of sentinel lymph nodes (SLNs) removed in.

**Methods:**

We searched all relevant studies published between January 2000 and October 2019. All data on for evaluation of SLN detection rate, number of SLNs removed per patient, and tumor positive rate of SLNB were extracted.

**Results:**

A total of 30 studies, including 4,216 SLN procedures were retrieved. There was a statistically significant difference between ICG and BD method in terms of SLN detection rate (OR, 6.73; 95% CI, 4.20-10.78). However, there was no significant difference between ICG and RI in this regard (OR, 0.90; 95% CI, 0.40-2.03). The number of SLNs removed per patient were 2.35 (1.46-5.4), 1.92 (1.0-3.64), and 1.72 (1.35-2.08) for ICG, BD, and RI, respectively. Only in 8 studies, the tumor positive rates in SLNB could be analyzed (ICG, 8.5-20.7%; BD, 12.7-21.4%; RI, 11.3-16%).

**Conclusion:**

ICG-SLNB could be an additional or an alternative method for axillary node mapping in breast cancer.

## Introduction

Axillary management is one of the most challenging aspects of breast cancer surgery. In this regard, sentinel lymph node biopsy (SLNB) constitutes the standard care for patients with clinically negative axillary lymph nodes (Verbeek et al., 2014). Many studies showed the safety of sentinel lymph node management in overall survival, disease-free survival, and regional control in comparison with standard axillary lymph node dissection (Krag et al., 2010). 

There are 2 main types of injection materials widely used in SLNB; namely blue dye (BD) and radio-labeled isotope (RI). Depending upon the practitioner’s preference, either BD or RI can be used alone or in combination with each other. In a meta-analysis, it was found that the false-negative rate of BD was higher (8.6%) than that of RI (7.4%) and combination of them (5.9%) (Pesek et al., 2012). A panel of experts also recommended the combination technique for maximizing sentinel lymph node (SLN) detection rate and enhancing negative predictive value (Lyman et al., 2005). However, only BD is used in some institutions since the RI method requires a sophisticated technique for injection and lymphoscintigraphy. Additionally, patients are sometimes contraindicated for RI-SLNB.

As a de novo technique, indocyanine green (ICG) fluorescence SLNB method (ICG-SLNB) is increasingly used as an alternative method in many breast cancer centers. With this method, the SLN detection rate can reach up to 90% (Kitai et al., 2005). Although ICG-SLNB has been introduced for over a decade (Motomura et al., 2003; Kitai et al., 2005), it still has not been included in a standard guideline as an option for axillary management. Subsequent meta-analysis analyzed the detection rate of ICG-SLNB in many types of cancer, such as breast, colorectal, lung, cervical, gastric, prostate, esophageal, and oral cancers (Xiong et al., 2014). 

There was a meta-analysis which compared ICG with conventional material (Sugie et al., 2017). Most of the included studies considered RI-SLNB as a standard method. In addition, over the past decade, there were randomized controlled studies comparing ICG-SLNB with BD and RI method in respect of detection rates of SLNB, the number of SLNs removed, or tumor positive rate of SLNB. Consequently, the aim of our study was to conduct a systematic review and meta-analysis to compare ICG-SLNB with the conventional BD and RI methods.

## Materials and Methods

A systematic literature search was performed using PubMed and SCOPUS databases. All relevant studies published between January 1^st^, 2000 and October 31^st^, 2019 were included. The Medial Subject Heading (MESH) terms, including “indocyanine green”, “sentinel lymph node”, and “breast” were used as keywords during search on the databases. The inclusion criteria were as follows: (i) breast cancer with clinically lymph node negative patient; (ii) ICG guided and other modalities for SLNB mapping concurrently, (iii) the SLNB as major focus, and (iv) available pathological data. The included studies reported the detection rate of SLNB for each modality.

Reviews, meta-analyses, abstracts, letters, case report, case series, commentaries, or duplicated publications, articles with overlapped data , articles with unavailable pathological data, animal studies, studies on other cancers, and non-English published studies were excluded.

*Data extraction and quality assessment*

Data were extracted and checked by two researchers (S. Thongvitokomarn and N. Polchai). Data about articles’ first author, year of publication, sample size, and tracers were extracted. The following results allowed for meta-analysis: (a) “SLN detection rate”, defined as a total number of patients whose SLNs were detected by each tracer divided by a total number of patients in each tracer material, (b) “number of SLNs removed per patient”, defined as a total number of SLNs harvested by each tracer divided by a total number of patients whose SLNs were detected, and (c) “tumor positive rate of SLNB”, defined as a number of pathological positive SLNs divided by a total number of SLNs detected by each tracer. Due to the possibility of a complete pathological response in neoadjuvant patients, the “tumor positive rate of SLNB” was not collected in the neoadjuvant setting. All discrepancies were discussed and resolved by consensus of the researchers. 

The quality of cohort studies was evaluated using the guideline of the STrengthening the Reporting of Observational Studies in Epidemiology (STROBE). The Cochrane risk of bias tool was used for quality assessment of the randomized controlled studies (Supplement 1).

*Statistical analysis*

The Mantel-Haenszel fixed effects and random effects model were used to obtain odd ratio (OR) for SLNB detection in 2 comparative groups, ICG versus BD and ICG versus RI. The statistical heterogeneity among studies was calculated using I^2^ statistics and p-values. The heterogeneity was considered significant where I^2^>50% or p<0.05. Publication bias of all included studies was exhibited in funnel plot.

Data with respect to the number of SLNs removed and tumor positive SLNs from most studies were expressed as mean or median. As raw data and standard deviation were not available, meta-analysis in this aspect could not be performed.

## Results

**Figure 1:**
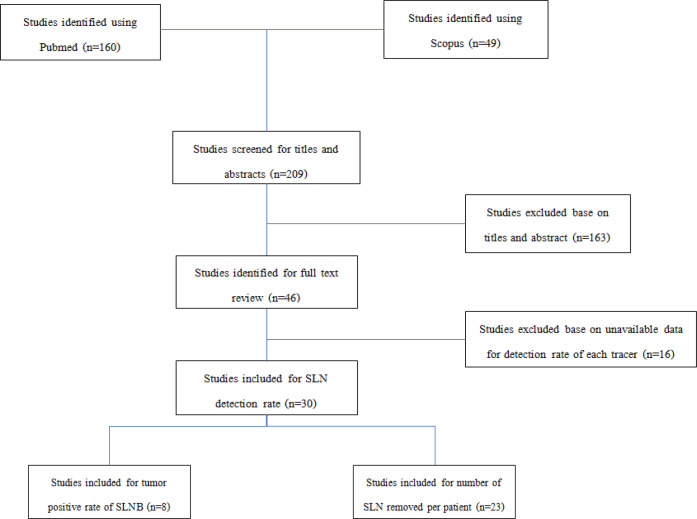
Diagram for Selection of the Included Studies

We initially identified a total of 160 potentially eligible studies from Pubmed datatbase and 49 additional studies from Scopus datatbase. Out of 209 relevant studies, 163 were excluded based on their titles or abstracts, i.e., not using standard method (BD and RI) concurrently, having review or commentary formats, using a language other than English, being animal studies, and studying f other cancers. Among the remaining 46 studies which could be retrieved in full text, 16 were excluded because of having no data on the detection rate of SLNs in each tracer. Consequently, 30 studies were considered involving 4,216 SLN procedures. Out of these 30 articles, 8 studies analyzed tumor positive rate of SLNB (Figure 1).

The characteristics and details of each study’s outcomes are summarized in Table 1. There were 26 cohort and 4 randomized controlled studies (one compared ICG with BD and others involved multimodal method comparison). The overall SLN detection rates using ICG, BD, and RI ranged from 69 to 100%, 65.6 to 97.1%, and 85 to 100%, respectively. The number of SLN removed per patient averaged 2.35 (ranging from 1.46 to 5.4) for ICG, 1.92 (ranging from 1.0 to 3.64) for BD,and 1.72 (ranging from 1.35 to 2.08) for RI (Table 2). With respect to studies with data eligible for analysis of tumor positive rate of SLNB, the result ranged from 8.5 to 20.7% for ICG, 12.7 to 21.4% for BD, and 11.3 to 16% for RI.

**Figure 2:**
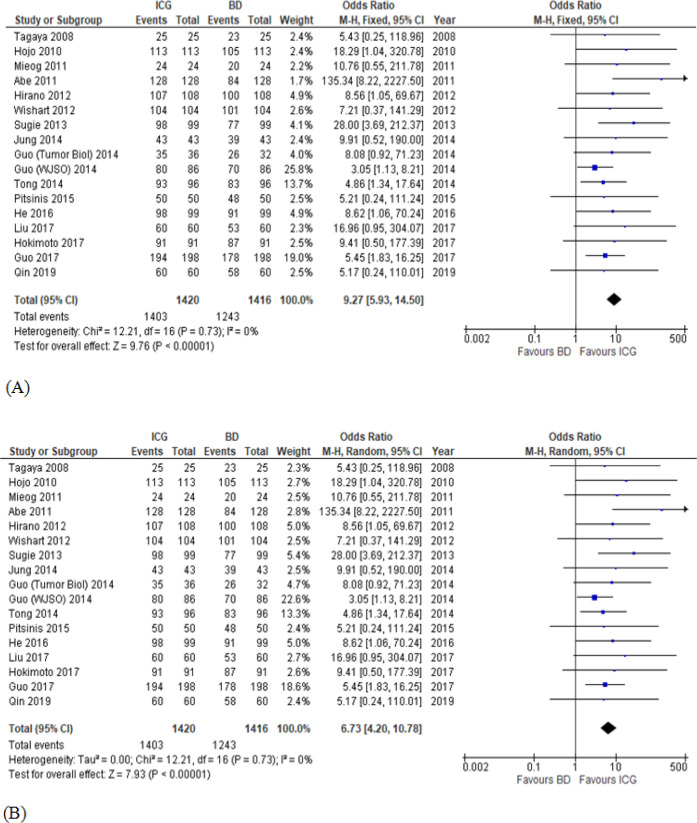
SLN Detection Rate (ICG versus BD); (A), fixed effects model; (B), random effects model

**Figure 3:**
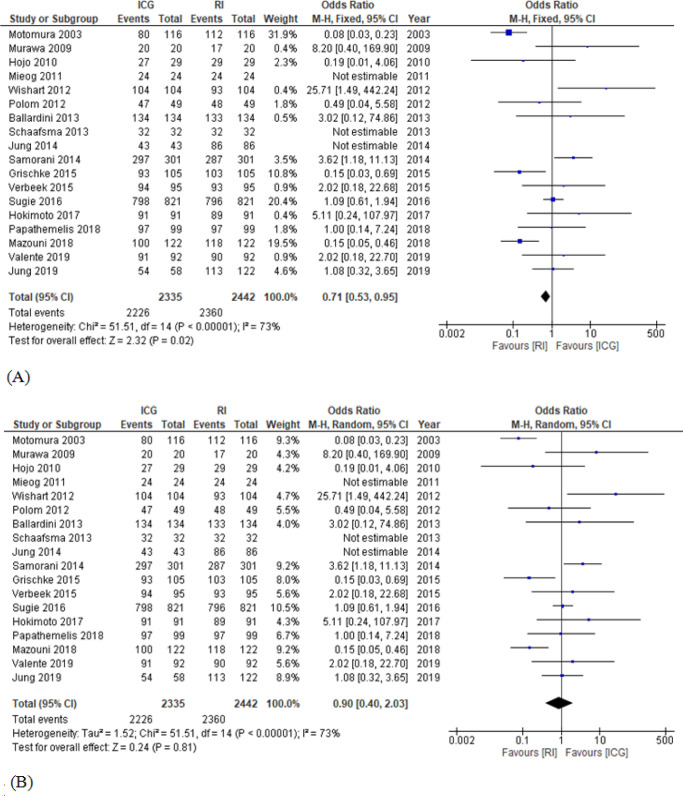
SLN Detection Rate (ICG versus RI); (A), fixed effects model; (B), random effects model

**Figure 4:**
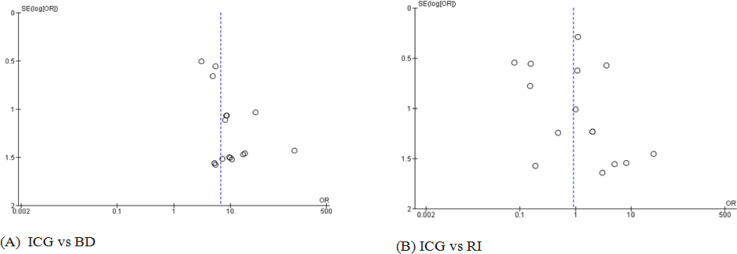
Funnel Plot of Included Studies in the Meta-Analysis; (A), 17 studies of SLN detection between ICG and BD; (B), 18 studies of SLN detection between ICG and RI

For meta-analysis, data for the SLN detection rate were categorized into 2 comparative groups; namely ICG versus BD and ICG versus RI. Odd ratio (OR) of SLN detection between ICG and BD and that of SLN detection between ICG and RI could be analyzed in 17 and 18 studies, respectively. There was a statistically significant difference in SLN detection rate between ICG and BD with respect to both fixed effects model (OR, 9.27; 95% confidence interval [CI], 5.93-14.50) and random effects model (OR, 6.73; 95% CI, 4.20-10.78). However, there was no heterogeneity of SLN detection rate between ICG and BD (I2, 0%; p, 0.73) (Figure 2). In an analysis for SLN detection rate between ICG and RI, there was a slight difference in fixed effect model which favored RI (OR, 0.71; 95% CI, 0.53-0.95). However, the result from random effects model was not significantly different (OR, 0.90; 95% CI, 0.40-2.03). The heterogeneity of SLN detection rate of the latter was observed with I2=73% and p< 0.00001 (Figure 3). According to the funnel plot, there was less publication bias in ICG versus RI group than ICG versus BD group (Figure 4). Due to the scarce resources of certain data, i.e., standard deviation, the meta-analysis in respect to the number of SLNs removed per patient and the tumor positive rate of SLNB could not be analyzed.

**Table 1:** Characteristics of 30 Included Studies

Authors and Year	Study	Tracer	N	SLN detection rate (%)	Tumor -positive rate of SLNB (%)
(Qin et al., 2019)	RCT	ICG+BD vs BD vs CN	ICG: 60 BD: 60	ICG: 60/60 (100%) BD: 58/60 (96.7%)	ICG: 17/199 (8.5%) BD: 13/102 (12.7%)
(Jung et al., 2019)	RCT	ICG+RI vs RI	ICG: 58 RI: 122	ICG: 54/58 (93.1%) RI: 113/122 (92.6)	N/A
(Valente et al., 2019)	Cohort	ICG+RI	92	ICG: 91/92 (98.9%) RI: 90/92 (97.8%)	ICG: 24/224 (10.7%) RI: 23/202 (11.3%)
(Mazouni et al., 2018)	Cohort	ICG+RI	122	ICG: 100/122 (81.9%) RI: 118/122 (96.7%)	ICG: 15/100 (15%) RI: N/A
(Papathemelis et al., 2018)	Cohort	ICG+RI	99	ICG: 97/99 (97.9%) RI: 97/99 (97.9%)	ICG: 27/215 (12.5%) RI: 24/172 (13.9%)
(Hokimoto et al., 2017)	Cohort	ICG+BD+RI	91	ICG: 91/91 (100%) BD: 87/91(95.6%) RI: 89/91 (97.8%)	N/A
(Guo et al., 2017)	Cohort	ICG+BD	198	ICG: 194/198 (98%) BD: 178/198(89.9%)	N/A
(Liu et al., 2017)	Cohort	ICG+BD	60	ICG: 60/60 (100%) BD: 53/60 (88.3%)	N/A
(He et al., 2016)	Cohort	ICG+BD	99	ICG: 98/99 (99%) BD: 91/99 (92.9%)	N/A
(Sugie et al., 2016)	Cohort	ICG+RI	821	ICG: 798/821 (97.2%) RI: 796/821 (97%)	N/A
(Pitsinis et al., 2015)	Cohort	ICG+BD	50	ICG: 50/50 (100%) BD: 48/50 (96%)	ICG: 18/87 (20.7%) BD: 18/84 (21.4%)
(Grischke et al., 2015)	Cohort	ICG+RI	105	ICG: 93/105 (88.6%) RI: 103/105 (98.1%)	N/A
(Samorani et al., 2015)	Cohort	ICG+RI	301	ICG: 297/301 (98.7%) RI: 287/301 (95.3%)	ICG: 70/583 (12%) RI: 55/458 (12%)
(Verbeek et al., 2014)	Cohort	ICB+RI+/-BD	95	ICG: 94/95 (98.9%) RI: 93/95 (97.9%)	ICG: 22/177 (12.4%) RI: 20/155 (12.9%)
(Tong et al., 2014)	Cohort	ICG+BD vs BD	96	ICG: 93/96 (96.9%) BD: 83/96 (86.5%)	N/A
(Guo et al., 2014b)	Cohort	ICG+BD	86	ICG: 80/86 (93%) BD: 70/86 (81.4%)	N/A
(Jung et al., 2014)	RCT	ICG+RI+BD vs RI	ICG+RI+BD:43 RI: 43	ICG: 43/43 (100%) BD: 39/43 (90.7%) RI: 86/86 (100%)	N/A
(Guo et al., 2014a)	RCT	ICG vs BD	ICG: 36 BD: 32	ICG: 35/36 (97.2%) BD: 26/32 (81.3%)	N/A
(Ballardini et al., 2013)	Cohort	ICG+RI	134	ICG: 134/134 (100%) RI: 133/134 (99.3%)	N/A
(Schaafsma et al., 2013)	Cohort	ICG+RI+BD	32	ICG: 32/32 (100%) RI: 32/32 (100%)	N/A
(Sugie et al., 2013)	Cohort	ICG+BD	99	ICG: 98/99 (99%) BD: 77/99 (77.8%)	N/A
(Hirano et al., 2012)	Cohort	ICG+BD vs BD	108	ICG: 107/108 (99.1%) BD:100/108 (92.6%)	N/A
(Wishart et al., 2012)	Cohort	ICG+RI+BD	104	ICG: 104/104 (100%) BD: 101/104 (97.1%) RI: 93/104 (89.4%)	ICG: 28/204 (13.7%) BD: 25/191 (13.1%) RI: 25/156 (16%)
(Polom et al., 2012)	Cohort	ICG+RI	49	ICG: 47/49 (95.9%) RI: 48/49 (98%)	N/A
(Mieog et al., 2011)	Cohort	ICG+RI+BD	24	ICG: 24/24 (100%) BD: 20/24 (83.3%) RI: 24/24 (100%)	N/A
(Abe et al., 2011)	Cohort	ICG+BD	128	ICG: 128/128 (100%) BD: 84/128 (65.6%)	N/A
(Hojo et al., 2010)	Cohort	ICG+BD vs ICG+RI	ICG+BD: 113 ICG+RI: 29	ICG1: 113/113 (100%) BD: 105/113 (92.9%) ICG2: 27/29 (93.1%) RI: 29/29 (100%)	N/A
(Murawa et al., 2009)	Cohort	ICG+RI vs ICG	20	ICG: 20/20 (100%) RI: 17/20 (85%)	N/A
(Tagaya et al., 2008)	Cohort	ICG+BD	25	ICG: 25/25 (100%) BD: 23/25 (92%)	N/A
(Motomura et al., 2003)	Cohort	ICG+RI	116	ICG: 80/116 (69%) RI: 112/116 (96.6%)	N/A

N/A, not applicable; SLNB, sentinel lymph node biopsy; ICG, indocyanine green; BD, blue dye; RI, radioisotope; RCT, randomize control trial

**Table 2:** Details of Included Studies with Number of SLNs Removed Per Patient

Author and Year	Number of SLNs removed per patient		
	ICG (mean)	BD (mean)	RI (mean)
(Qin et al., 2019)	199/60 (3.31)	102/60 (1.7)	N/A
(Jung et al., 2019)	127/54 (2.35)	N/A	232/113 (2.05)
(Valente et al., 2019)	224/91 (2.46)	N/A	202/90 (2.24)
(Papathemelis et al., 2018)	215/99 (2.17)	N/A	172/99 (1.73)
(Liu et al., 2017)	177/60 (2.95)	106/53 (2.00)	N/A
(He et al., 2016)	276/98 (2.82)	202/91 (2.22)	N/A
(Sugie et al., 2016)	1835/798 (2.30)	N/A	1353/796 (1.70)
(Pitsinis et al., 2015)	87/50 (1.74)	84/48 (1.75)	N/A
(Grischke et al., 2015)	138/93 (1.48)	N/A	157/103 (1.52)
(Verbeek et al., 2014)	177/94 (1.88)	N/A	155/93 (1.67)
(Samorani et al., 2015)	583/297 (1.96)	N/A	458/287 (1.60)
(Guo et al., 2014b)	281/80 (3.51)	255/70 (3.64)	N/A
(Guo et al., 2014a)	126/35 (3.60)	54/26 (2.10)	N/A
(Ballardini et al., 2013)	245/134 (1.83)	N/A	231/133 (1.74)
(Schaafsma et al., 2013)	48/32 (1.50)	N/A	48/32 (1.50)
(Sugie et al., 2013)	281/98 (2.87)	121/77 (1.57)	N/A
(Hirano et al., 2012)	235/107 (2.20)	160/100 (1.60)	N/A
(Wishart et al., 2012)	204/104 (1.96)	191/101 (1.89)	156/93 (1.68)
(Polom et al., 2012)	113/47 (2.40)	N/A	100/48 (2.08)
(Mieog et al., 2011)	35/24 (1.46)	30/20 (1.50)	35/24 (1.46)
(Abe et al., 2011)	397/128 (3.10)	84/84 (1.0)	N/A
(Murawa et al., 2009)	35/20 (1.75)	N/A	23/17 (1.35)
(Tagaya et al., 2008)	135/25 (5.40)	53/23 (2.30)	N/A
Total SLNs per patient (mean)	6,173/2,628 (2.35)	1,440/753 (1.92)	3,322/1,928 (1.72)

N/A, not applicable; SLN, sentinel lymph node; ICG, indocyanine green; BD, blue dye; RI radioisotope

## Discussion

SLNB is a technique which identifies SLNs containing cancer cells which should be removed in patients with breast cancer. This mapping technique currently has an essential role for the disease’s staging, surgical treatment, and prognosis. Since the diagnostic tool technically helps surgeons to find out cancer in SLNs, tracer materials are required to be injected into the breast for such detection. The conventionally used materials for the detection of SLNs are BD and RI. Although utilizing BD is very convenient and inexpensive, some patients undergoing BD might experience allergic reactions or hypotension (Bezu et al., 2011). 

The detection rate was reportedly enhanced by using RI in lieu of BD. In addition, the false-negative rate was reduced when RI instead of BD was used. Moreover, the combination of BD and RI resulted in the lowest false-negative rate (Pesek et al., 2012). In ALMANAC trial, SLN identification rate was 85.6%, 85.6%, and 96% when BD, RI, and combination of BD and RI were respectively used (Latosinsky et al., 2008). However, since the RI method requires a sophisticated technique for injection and lymphoscintigraphy, its implementation is limited in some institutions. 

ICG has a short half-life in plasma. It strongly binds to plasma bilirubin and is absorbed to lymphatic vessels immediately. Given that the ICG functions as a fluorescent tracer appearing on near-infrared imaging system, it provides a real-time visualization which helps surgeons decide the precise location of the skin incision. Nevertheless, the implementation of ICG has some limitations, for example, the leakage of the tracer occurs during harvesting SLNs. The spreading of the material results in difficulty in SLN identification.

Recent meta-analyses showed that ICG-SLNB in breast cancer had a high detection rate for SLN and was reliable for the detection of SLN metastasis (Xiong et al., 2014). In a meta-analysis by Zhang et al., 19 studies with 2,594 breast cancer patients were included. The results showed that ICG-guided SLN detection rate was 98% (95% CI 0.96-0.99) and the sensitivity was 92% in diagnostic performance in the presence of metastases (95% CI 0.85-0.96) (Zhang et al., 2016).

The detection rate was reportedly enhanced when the combination was used (Guo et al., 2017; Vermersch et al., 2019). In aforementioned study, it was found that SLN detection rates with ICG, BD, and their combination were 97%, 89%, and 99.5%, respectively (p<0.001). Their combination also resulted in more lymph node identification per patient (median 3 versus 2 nodes). They also revealed that their combination would reduce false-negative rate of SLN detection from 12 to 4% (Guo et al., 2014b).

In addition, ICG improved detection rates of SLNs when it was combined with RI (98.6 versus 95.3%) with the concordance index of both methods of 98.75% (95% CI, 97.1-99.5) (Samorani et al., 2015). Although the overall detection rate of SLNs was identical to that of RI (97.2 versus 97%, p=0.88) when ICG was used in a study investigated 821 patients, their combination achieved higher improvement compared with when only RI was used (99.8 versus 97%, p<0.001) (Sugie et al., 2016). The authors also found that the detection rate of metastatic SLNs was the highest when combination of ICG and RI (97.2%) were used compared to ICG alone (93.3%) and RI alone (90%). 

ICG-SLNB was also reported in the neoadjuvant setting in a previous study (Jung et al., 2019). The authors of aforementioned study reported the safety of ICG-SLNB combined with RI-SLNB in these group of patients. This is an interesting issue as the axillary dissection is still mandate for the preoperative node positive patients who receive neoadjuvant systemic treatment. The ICG-SLNB can be further evaluated for detection rate and false-negative rate in this setting.

Our meta-analysis aimed to compare diagnostic performance of ICG with standard tracers (both BD and RI) regarding SLNB in breast cancer patients. This meta-analysis yielded no statistically significant difference between ICG and RI for SLN detection rate in the random effect model, implying that ICG-SLNB is a reliable tool for SLN detection compared with the standard RI method. In contrast, the detection rate was significantly different between ICG and BD. 

We also demonstrated a higher number of SLNs removed per patient in ICG-SLNB than that of standard methods. Whether the increased number of SLNs yielded by ICG-SLNB is secondary to adjacent lymph nodes staining from ICG leakage or ICG-SLNB renders a better axillary staging when compared with the standard methods needs further investigation. As the detection rate cannot predict the tumor positive rate of SLNB, the false-negative rate must be considered. Mok et al., (2019)reported the pooled estimated false-negative rate at 0.6% (-0.3,1.5) for ICG-SLNB. The authors also reported the false-negative rate of RI-SLNB at 2.6 (0.7,4.6). The false-negative rate could not be evaluated in many studies as the standard treatment of negative SLNB patients is omission of further axillary dissection. 

The result from this study supported the use of ICG. The detection rate and number of SLNs removed from ICG proved the safety and feasibility of this technique. In the setting that RI is not available, ICG-SLNB is a good diagnostic tool for SLNB combined with BD method. However when the RI-SLNB is available, ICG-SLNB should be considered as an alternative method. For instance, when the surgeon cannot identify the SLN from RI preoperatively, the ICG should be added concurrently with BD-SLNB. In contrast, if the patient is pregnant, the surgeons should avoid BD-SLNB as it can cause anaphylaxis. NCCN guideline discourages the use of BD-SLNB in pregnant women. For using ICG-SLNB in pregnant women, there are many studies in both human and animal models on using ICG during surgical procedure. In the past, ICG was used for the measurement of liver blood flow and cardiac output in pregnant women (Robson et al., 1990). There were reports showing that ICG could not cross the placenta. The researchers could not detect the ICG in fetal blood (Probst et al., 1970; Rudolf et al., 1977). Additionally, there was also a study showing the protective effect of the placenta after ICG injection on the ex-vivo perfusion model (Rubinchik-Stern et al., 2016). Gynecologists also used ICG for lymph node mapping for pregnant cervical cancer patients. The patients and their babies were all healthy after the procedure and the delivery (Abe et al., 2011). Moreover, there was also a report showing the use of ICG in pregnant patients who needed ophthalmic evaluations (Fineman et al., 2001). ICG is pregnancy category C which means that the animal study has never been conducted and the fetal harm cannot be demonstrated. Conclusively, maintaining the dual technique SLNB can be achieved in pregnant women.

The difficulty during the ICG-SLNB should be concerned. As the ICG cannot be visualized directly, some surgeons experienced the problems of identifying the SLNs from the screen. Chang reported the use of blue light-emitting instead of near-infrared light (Chang et al., 2019). They reported the detection rate of blue-light SLNB at 93.4%. 

There were some limitations in our study. The data regarding long-term follow-up for ICG-SLNB were insufficient. The difference in sample size, ranging from 24 to 821, possibly resulted in bias in our study. Finally, the different outcomes might be due to various techniques performed in each study, such as using different imaging systems, ICG dose, and time of injection to skin incision .

In conclusion, based on the results of this systematic review and meta-analysis, ICG-SLNB had better detection rate than BD-SLNB alone. An insignificant difference in detection rate compared to RI-SLNB was also found. Conclusively, ICG-SLNB can be either an additional method or a replacement method for axillary node staging. However, the surgical technique and operative detail must be standardized, and the surgeon should be familiar with it. 
